# Post-Hysterectomy Vaginal Vault Prolapse: Current Perspectives and Decision-Making Considerations for Surgical Treatment

**DOI:** 10.3390/medicina62081540

**Published:** 2026-08-11

**Authors:** Sveto Pantovic, Jelena Dotlic, Katarina Ivanovic, Kosta Pantovic, Milica Jeremic, Milena Mitrovic, Ivana Vukovic, Branislav Milosevic, Milos Milincic, Stefan Ivanovic, Slavica Aksam

**Affiliations:** 1 Clinic of Obstetrics and Gynecology, University Clinical Center of Serbia, Dr Koste Todorovica 26, 11000 Belgrade, Serbiaikatarina.1996@gmail.com (K.I.); pantovick96@gmail.com (K.P.); milosmilincic@gmail.com (M.M.); 2 Medical Faculty, University of Belgrade, Dr Subotica 8, 11000 Belgrade, Serbia; 3 Clinic of Obstetrics and Gynecology “Narodni Front”, Kraljice Natalije 62, 11000 Belgrade, Serbia

**Keywords:** post-hysterectomy, vaginal vault prolapse, vaginal eversion, surgical treatment, outcome

## Abstract

*Background and Objectives:* Following the removal of the uterus, due to prolapse or other indications, disruption of the apical support structures may result in descent of the vaginal apex. As the condition progresses, the cystocele, rectocele, and/or enterocele are formed, finally creating a complex vaginal eversion that generally requires surgical multi-compartmental repair. This review aimed to provide a comprehensive overview of cur-rent surgical modalities for post-hysterectomy vaginal vault prolapse, which could sup-port personalized treatment. *Materials and Methods:* MEDLINE, Scopus and Google Scholar databases were searched to retrieve freely available manuscripts published in English language since the year 2000. *Results:* Surgical correction remains the basis of management for symptomatic post-hysterectomy vaginal vault prolapse. The primary objective of treatment is restoration of robust apical support, normalization of vaginal axis, preservation of sexual and urinary function along with minimization of operative complications and recurrence. Several reconstructive approaches have been established as safe and comparatively reliable. They are classified into native tissue vaginal repairs (sacrospinous ligament fixation, uterosacral ligament suspension), abdominal or minimally invasive sacro-colpopexy, mesh-augmented techniques, and obliterative procedures. No single surgical approach appears universally superior across anatomical, functional, and safety outcomes, and procedure selection should therefore be individualized according to patient characteristics, treatment priorities, and surgeon expertise. Consequently, procedure selection should be individualized according to patient characteristics and surgeon expertise. *Conclusions:* Post-hysterectomy vaginal vault prolapse remains a complex and clinically significant condition. Current evidence supports the central importance of apical suspension during all hysterectomies. Optimal treatment outcomes are achieved through patient-centered decision-making that balances durability, safety, functional recovery, and individual expectations.

## 1. Introduction

Pelvic organ prolapse (POP) is a common benign gynecologic condition that affects up to 50% of parous women, out of whom 10–20% will eventually need surgical treatment [1,2,3]. One of the most complex and challenging POP conditions is post-hysterectomy vaginal vault prolapse. It occurs after approximately 5–20% of hysterectomies. Its incidence increases with advancing age and varies according to indications and surgical approach of performed hysterectomy. The prevalence of vaginal vault prolapse after hysterectomy performed due to non-prolapse indications is approximately 4.4% for laparoscopic and 5.8% for vaginal hysterectomy. In contrast, rates of up to 23% have been described following vaginal hysterectomy performed for uterine prolapse [4,5,6].

Following hysterectomy, disruption of the apical support structures may result in descent of the vaginal apex [2]. The severity of pelvic organ prolapse is currently assessed using the Pelvic Organ Prolapse Quantification (POP-Q) system, which represents the internationally accepted standard for objective clinical assessment and reporting of prolapse. According to the POP-Q classification, prolapse is categorized into stages 0–IV based on the position of the leading edge relative to the hymen [7,8].

As prolapse progresses, associated cystocele, rectocele, and/or enterocele may develop, ultimately resulting in complex multicompartment prolapse that frequently requires combined surgical repair [9,10]. Complex vaginal eversion, characterized by apical prolapse associated with prolapse of both the anterior and posterior vaginal compartments, has been reported in up to 67% of high-risk patients, including apical prolapse associated with cystocele in 7% of cases, rectocele in 30%, and combined cystocele and rectocele in 30% [5,6,11].

Vaginal vault prolapse substantially impairs women’s quality of life. In addition to vaginal bulging, affected women frequently report pelvic pressure, vaginal ulceration, urinary incontinence, defecatory dysfunction, dyspareunia, and sexual dissatisfaction [4,5,12]. In advanced stages, the condition may significantly compromise patient’s daily activities, self-esteem, and social functioning [6,13]. Such patients impose a considerable economic burden on healthcare through recurrent interventions, prolonged recovery, and chronic symptoms [12].

Numerous surgical techniques have been developed over the past several decades to address post-hysterectomy vaginal vault prolapse [1,2]. These include sacrospinous ligament fixation, uterosacral ligament suspension, abdominal or minimally invasive sacrocolpopexy, and mesh-augmented vaginal repairs. Despite considerable advances in reconstructive pelvic surgery, there is still no universal consensus regarding the optimal surgical approach [9,14,15]. Owing to their shorter operative time and lower perioperative morbidity, transvaginal native tissue repairs remain among the most frequently performed procedures. Nevertheless, their long-term efficacy varies across patient populations and published studies [16]. Abdominal sacrocolpopexy and its laparoscopic and robotic modifications provide durable anatomical support but are associated with longer recovery and greater resource utilization [17]. At the same time, concerns regarding mesh-related complications and subsequent regulatory restrictions on transvaginal mesh have renewed interest in native tissue reconstruction [18,19,20].

Given the heterogeneity of patient characteristics, prolapse severity, recurrence status, previous pelvic floor surgery, sexual activity, comorbidities, and surgeon expertise, selecting the most appropriate treatment remains one of the greatest challenges in the management of post-hysterectomy vaginal vault prolapse. Although existing narrative reviews and clinical guidelines summarize the available surgical options, they provide limited practical guidance regarding individualized treatment selection in women with recurrent, multicompartment, or medically complex prolapse [9,14,15].

Therefore, this review aims to summarize the current evidence regarding the surgical management of post-hysterectomy vaginal vault prolapse while providing a clinically oriented framework to support individualized therapeutic decision-making in everyday practice. Unlike previous reviews that primarily describe individual surgical techniques, the present review emphasizes clinically relevant decision-making by integrating patient characteristics, prolapse severity, recurrence status, sexual function, frailty, comorbidities, previous pelvic floor surgery, mesh-related considerations, patient-reported outcomes, and surgeon expertise.

## 2. Materials and Methods

### 2.1. Literature Search

This review was designed as a narrative literature review based on a structured search of the existing evidence.

A literature search was performed using the PubMed/MEDLINE, Scopus, and Web of Science databases to identify the most relevant publications addressing the surgical management of post-hysterectomy vaginal vault prolapse. The search strategy was organized around thematic groups of keywords covering different surgical approaches, treatment options, clinical outcomes, and factors influencing surgical decision-making.

The following search terms were used individually and in various combinations: *post-hysterectomy vaginal vault prolapse, vaginal vault prolapse, apical prolapse, pelvic organ prolapse, sacrocolpopexy, laparoscopic sacrocolpopexy, robotic sacrocolpopexy, sacrospinous ligament fixation, uterosacral ligament suspension, native tissue repair, transvaginal mesh, pectopexy, vNOTES, colpocleisis, pessary, recurrence, complications, quality of life,* and *decision-making*.

### 2.2. Literature Selection Criteria

This narrative review included publications providing clinically relevant information on the surgical treatment of post-hysterectomy vaginal vault prolapse in women, treatment outcomes, complications, recurrence, quality of life, and factors influencing the choice of the most appropriate surgical approach.

Accessible manuscripts written in English and published between January 2010 and May 2026 were considered eligible. Both primary studies and secondary literature (systematic reviews, meta-analyses, and clinical guidelines) were included.

Priority was given to more novel data, higher-level evidence such as original research articles, high-quality observational studies, international society guidelines, and consensus statements, but when it was unavailable or insufficient, all studies that provided clinically meaningful information were included.

### 2.3. Data Analysis and Synthesis

Titles and abstracts of studies found using the search strategy were screened independently by team members to identify manuscripts that potentially meet the review aims. After removal of unreliable, duplicate, and irrelevant records the full text of potentially eligible articles was retrieved.

These publications were analyzed qualitatively by team members independently. Any disagreement was resolved through discussion with supervising members until consensus was reached. Particular attention was given to the study population, type of prolapse, indications for individual surgical procedures, their advantages and limitations, anatomical and functional outcomes, recurrence rates, perioperative and long-term complications, patients’ quality of life, and recommendations issued by relevant international professional societies. Upon thorough assessment publications that were not directly related to the review aim or did not provide clinically meaningful information were excluded from the final analysis.

The data extracted from articles was synthesized through a thematic narrative approach to critically appraise the available evidence and relate it to contemporary clinical decision-making. The results of the research have been divided into different sections and subsections to illustrate what has been re-ported on the topic of the discussion.

**Chart 1:**
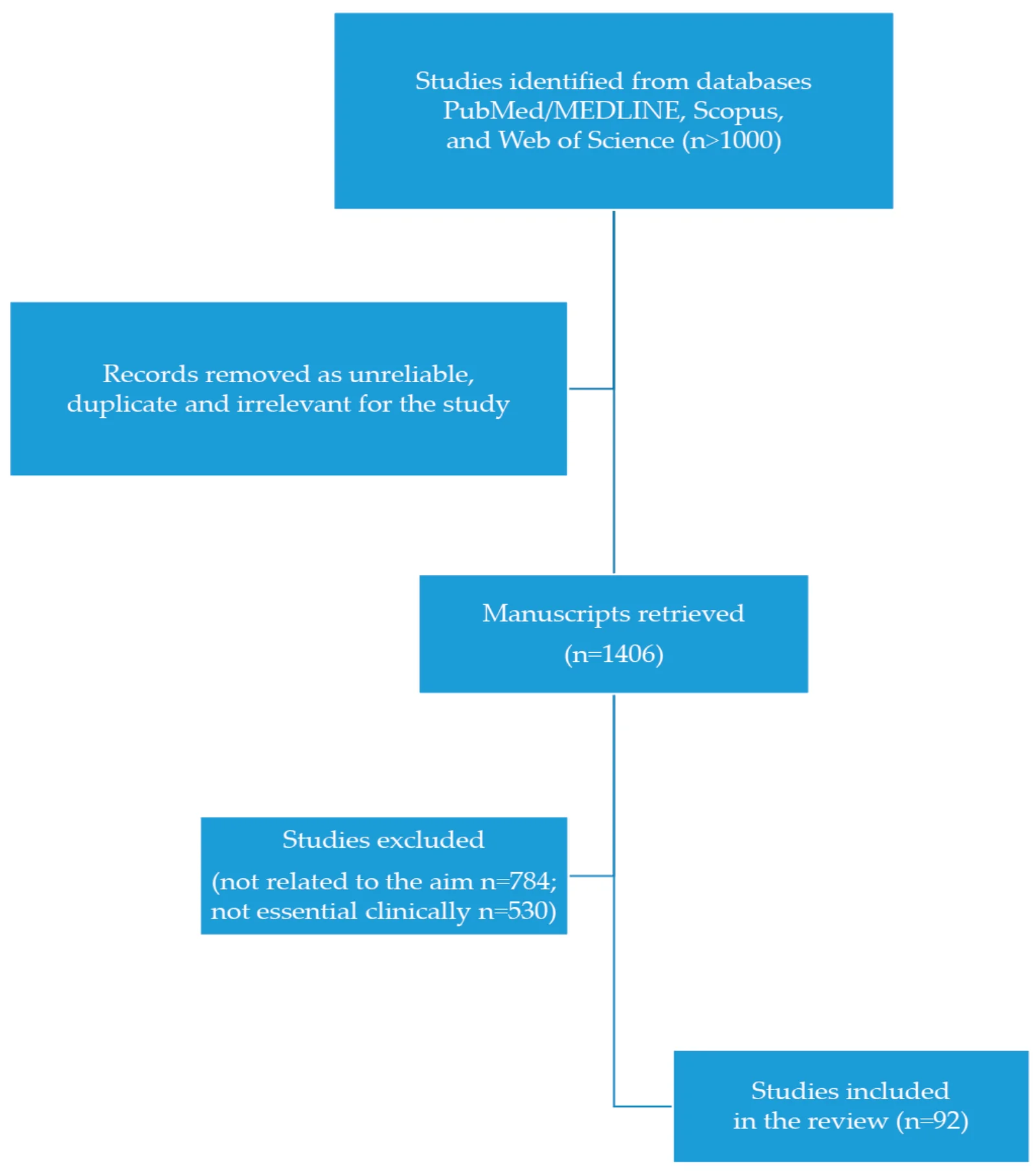
Flow diagram illustrating the literature selection process.

The identification, screening, and selection process is presented in the Chart 1 constructed solely to provide a transparent overview of the literature selection process rather than to classify this work as a systematic review.

### 2.4. Methodological Considerations and Limitations

Several limitations should be acknowledged. First, only English language manuscripts were included. Second, long-term functional outcomes such as sexual health and patient satisfaction after different surgical corrections of vaginal vault prolapse remain underreported in the literature as consequently were not included in this study.

Moreover, this manuscript was designed as a narrative review and although major studies and guidelines were included, formal systemic review or meta-analytic pooling was not performed. A narrative approach was considered more appropriate because of the substantial heterogeneity of the available literature, including differences in study design, investigated patient populations, surgical techniques, definitions of anatomical and functional success, recurrence criteria, duration of follow-up, and outcome reporting. Existing evidence includes randomized and non-randomized comparative studies, retrospective cohorts, single-center surgical series, guidelines, and review articles. Given this diversity in data and studies, a narrative approach was considered the most appropriate method to highlight current uncertainties affecting therapeutic decision-making.

Additionally, as studies regarding post-hysterectomy vaginal vault prolapse with Grade “A” evidence are relatively insufficient in available literature, we decided to include all studies (grade A–C) that met the inclusion criteria. The review aim was not to quantitatively pool the available evidence or estimate the overall effect of individual surgical procedures, but rather to critically evaluate and integrate the most relevant evidence in order to clarify its role in contemporary clinical decision-making.

The main limitations of this approach include the potential for selection bias during study inclusion, the absence of a formal risk-of-bias assessment, and the inability to perform statistical comparisons because of the methodological and clinical heterogeneity of the available studies. These limitations were addressed by conducting a structured literature search, applying predefined selection criteria, and independently reviewing the literature by multiple researchers upon which recommendations were made with the consent of all team members.

## 3. Anatomy and Pathophysiology

Normal vaginal support is maintained by the endopelvic fascia, ligamentous attachments, and pelvic floor musculature, which together provide dynamic and static stabilization of the pelvic organs. According to the DeLancey classification, pelvic support is organized into three anatomical levels, disruption of which represents the principal mechanism underlying pelvic organ prolapse development [8,21].

Level I support is provided by the cardinal uterosacral ligament complex and the paracolpium, which suspend the vaginal apex to the sacrum and lateral pelvic walls. Level II support involves the lateral attachment of the mid-vagina to the arcus tendineus pelvic fascia and levator ani fascia. This level prevents anterior (cystocele) and posterior (rectocele) wall defects. Level III support includes the perineal body, perineal membrane, and distal vaginal attachments that maintain introitus stability and prevent distal vaginal descent [8,22,23].

Disruption of the level I apical support results in descent of the vaginal cuff and leads to post-hysterectomy vaginal vault prolapse while the disruption of all three levels causes vaginal eversion [21,22].

After hysterectomy, especially when uterosacral or cardinal ligaments are inadequately reattached to the vaginal cuff, level I support may be compromised. Loss of apical suspension permits downward displacement and posterior rotation of the vaginal axis. Postoperative increases in intra-abdominal pressure caused by the daily activities of the patient along with aging and hormonal changes, progressively stretch the anterior and posterior compartments, resulting in multi-compartment prolapse [8,24].

Importantly, post-hysterectomy vaginal vault prolapse differs pathophysiologically from isolated cystocele or rectocele, as the primary defect is apical rather than fascial. Failure to address the apex during initial surgery has been consistently associated with higher recurrence rates indicating that restoration of apical support is essential for pre-venting post-hysterectomy prolapse and accompanying complications [9,25].

## 4. Risk Factors and Natural History

The development of post-hysterectomy vaginal vault prolapse is multifactorial [5]. It is caused by interactions between patients’ characteristics, such as age, body composition, connective tissue features, and obstetric history, as well as factors related to their previous surgery. The literature data show that the risk for POP and post-hysterectomy vaginal eversion increases with advancing age, obesity, parity, and chronic elevations of intra-abdominal pressure, such as chronic cough, constipation, and heavy physical activity [26,27].

Vaginal childbirth (multiparity, prolonged or complicated delivery, a large child or multifetal pregnancy, delivery with application of forceps/vacuum) remains one of the major risk factors for POP. Levator ani muscle trauma and fascial disruption during delivery can lead to the long-term weakening of the pelvic floor support structures [11,28].

Aging and menopause are also well-established POP risk factors particularly in women with additional risk factors including previous hysterectomy. Menopause-related hormonal changes may accelerate the progression of apical prolapse through several mechanisms. It is well known that estrogen receptors are distributed throughout the pelvic floor tissues. After menopause, estrogen deficiency contributes to altered collagen metabolism, reduced collagen synthesis and increased matrix degradation which can all lead to vaginal tissue atrophy and decreased tensile strength of pelvic connective tissue. Besides reduced ligamentous strength, hypoestrogenism can cause progressive weakening of muscular support, which may further compromise Level I suspension [27,29].

Hysterectomies themselves are a significant risk factor for subsequent vault prolapse. Vault prolapse occurs most often after hysterectomies that are performed due to uterine prolapse or when apical ligaments are not adequately reattached to the vaginal cuff at the time of the surgery [22]. In a systematic review and meta-analysis including more than 3.5 million women, hysterectomy was associated with an increased likelihood of pelvic organ prolapse beyond 10 years after surgery. The subsequent risk appears to be modified by the original indication for hysterectomy, pre-existing prolapse, parity, obesity, menopausal status, and the surgical approach. These findings underscore the importance of preserving or restoring adequate apical support at the time of hysterectomy, particularly in women at increased risk of subsequent prolapse [30,31].

However, in some other investigations no significant associations between the specific surgical techniques of the initial hysterectomy and the occurrence of vaginal vault prolapse after hysterectomy were confirmed. These studies found that the pre-operative POP stage and repeated prolapse repairs were the most important predictors of vault prolapse recurrence even if apical suspension was performed [32,33,34].

Addressing these risk factors is essential for the individualized choice of reconstructive techniques as well as for counseling patients on their prognosis and recurrence risk. Prolapse recurrence generally involves the vaginal vault and anterior compartment (10–20%). However, if it is not addressed in time, it will progress into complete vaginal eversion [6,24]. The literature data show that although numerous women develop vault prolapse after having hysterectomies, a smaller proportion will be symptomatic. Generally, only these patients will be the ones that will ultimately require surgical correction of the vaginal eversions [4,35].

## 5. Surgical Treatment Modalities

Surgical correction remains the basis of management for symptomatic post-hysterectomy vaginal vault prolapse. The primary treatment objectives are the restoration of robust apical support, the normalization of the vaginal axis, the preservation of sexual and urinary function, and the minimization of operative complications and recurrence [1,9]. Several reconstructive approaches have been established as safe and comparatively reliable. These approaches are generally classified into transvaginal that include 1. native tissue vaginal repairs (sacrospinous ligament fixation, iliococcygeus suspension and uterosacral ligament suspension), 2. abdominal or minimally invasive (sacrocolpopexy and uterosacral ligament suspension), 3. mesh-augmented techniques, and 4. obliterative procedures. According to studies from the literature, no single approach has proven to be superior in its effectiveness or lack of complications. Procedure selection should thus be individualized according to patient characteristics and surgeon expertise [2,25,33]. Table 1 presents the comparative outcomes and functional results of the currently used surgical methods for the management of symptomatic post-hysterectomy vaginal vault prolapse.

### 5.1. Native Tissue Vaginal Repairs

Sacrospinous ligament fixation (SSLF) is one of the most frequently performed vaginal procedures for repairing apical vaginal prolapse and restoring the anatomic posterior inclination position. In this procedure, the vaginal apex is anchored to the sacrospinous ligament [16,36]. This procedure either uses a special minimally invasive device or simple resorbable and non-resorbable sutures through the transvaginal posterior. Specialized devices and instruments that may facilitate suture placement during sacrospinous ligament fixation include the Miya hook, Arthrotek suture punch, Capio device, i-Stitch, Endostitch, Deschamps ligature carrier, aneurysm needle, Caspari device, and Breisky–Navratil retractors [17,37]. The route of dissection to the ligament is commonly through posterior vaginal wall. Alternatively, but less often, the anterior dissection can also be performed [17,36]. Investigations have shown that the cure rates for this procedure range between 80% and 90%, with significant symptom relief and high rates of patient satisfaction [6,34,36]. This procedure also offers several other advantages. These include short operative time, minimal blood loss, avoidance of abdominal surgery and use of synthetic materials, rapid recovery, and preservation of vaginal length and sexual function. Comparative studies demonstrate that SSLF provides similar anatomic outcomes to other native tissue techniques while maintaining favorable postoperative sexual function [13,16]. Compared to other vaginal operations, this type of surgery was also found to have lower risks in several areas. These include recurrent prolapse and consequently low reoperation rates as well as lower occurrence of postoperative urinary stress incontinence and dyspareunia. According to recent data, sacrospinous ligament fixation is thus a method of choice for the treatment of vaginal vault prolapse in over 75% of cases [38,39]. SSLF is usually applied unilaterally, but it can also be applied bilaterally, especially in patients who are at high risk of recurrent prolapse [40].

Despite all favorable outcomes, SSLF is considered a technically demanding procedure with a substantial learning curve, as safe suture placement within the deep pelvis requires detailed knowledge of pelvic anatomy and considerable surgical experience to minimize neurovascular and visceral complications [41].

Potential perioperative complications include vascular injury and hemorrhage (around 5%), rectal injury (0.5%), uretral injury (1%), and pudendal or sciatic nerve injury (up to 2%). Additionally, late complications include temporary pudendal or sciatic nerve irritation, fistulas, changes in vaginal anatomy (shortened, narrowed, disturbed ax-is), dispareunia (up to 16% in the first year), and chronic pain in the pelvis and legs (2%). However, the reported overall complication rates remain low, not exceeding 5% [34,36].

Recurrent vaginal vault prolapse after sacrospinous ligament fixation occurs in up to 19% of cases. These prolapses mostly occur in the anterior compartment due to the post-operative vaginal retroversion making the anterior vaginal wall more exposed to abdominal pressure. However, recurrent rectocele and urethral syndrome have also been reported [36]. To prevent recurrence, simultaneous treatment of the anterior wall is thus suggested for every colpopexy. It is also essential for high-risk patients, such as those who are older or obese or who participate in physically demanding daily activities [42]. Bilateral fixation may reduce vaginal axis deviation and dyspareunia compared to unilateral approaches, and it may also improve functional outcomes in selected patients. However, it increases the risk of rectum compression and thus is less commonly performed [38,39,40].

Uterosacral ligament suspension (USLS) restores apical support by attaching the vaginal cuff to the uterosacral ligaments. It can be performed either transvaginally or laparoscopically. This technique recreates physiological support while preserving normal vaginal orientation [43]. Its success rates are comparable to those of SSLF, at approximately 75–90%. Its reported recurrence rates are also similar to those of SSLF, at 10–20%. Again, this recurrence predominantly affects the anterior vaginal wall [32,44]. Comparative studies have shown that laparoscopic high uterosacral ligament suspension provides anatomical and functional outcomes comparable to laparoscopic sacrocolpopexy in appropriately selected patients, while maintaining the advantages of native tissue repair [45]. However, the proximity of the ureters increases the risk of ureteral kinking or injury. This necessitates intraoperative cystoscopy or ureteral visualization and, occasionally, assistance from urological surgeons [43,44].

A well-established modification of uterosacral ligament suspension is the Shull technique, in which the vaginal cuff is suspended bilaterally to the proximal uterosacral ligaments under direct visualization, thereby restoring physiological apical support while minimizing ureteral injury through routine intraoperative cystoscopic assessment. Long-term studies have demonstrated favorable anatomical and functional outcomes, making this technique an effective native tissue option for vaginal vault prolapse repair [46].

SSLF and USLS are frequently performed concurrently with hysterectomies as a prophylactic measure to prevent future vault prolapse. Additionally, native tissue vaginal repairs demonstrate consistently favorable outcomes for durable anatomical corrections and quality of life [32,34].

McCall culdoplasty is a native tissue procedure most commonly performed at the time of vaginal hysterectomy to prevent post-hysterectomy vaginal vault prolapse. The technique is based on obliteration of the posterior cul-de-sac and incorporation of the uterosacral ligaments into the vaginal cuff closure, thereby restoring apical support and reducing the risk of subsequent enterocele formation. It is particularly useful as a prophylactic procedure in women undergoing vaginal hysterectomy for stage I–III prolapse, whereas more advanced vault descent or complete vaginal eversion usually requires a formal apical suspension procedure. Modified McCall techniques, including incorporation of the uterosacral–cardinal ligament complex and cul-de-sac peritoneum, have also been described as effective approaches for supporting the vaginal apex while preserving vaginal length and minimizing postoperative morbidity [2,37,47].

Iliococcygeus suspension is another vaginal native tissue repair in which the vaginal apex is attached bilaterally to the iliococcygeus fascia or muscle. This procedure was developed as an alternative to sacrospinous ligament fixation, particularly because fixation to the iliococcygeus fascia avoids direct proximity to the pudendal neurovascular bundle and ureter. It may therefore be useful in patients with a short vagina, restricted vaginal mobility, or when access to the sacrospinous or uterosacral ligaments is limited. Compared with sacrospinous fixation, iliococcygeus suspension may better preserve a more central vaginal axis, although potential disadvantages include vaginal shortening, recur-rent prolapse, and lower vertical elevation of the apex in selected cases. According to literature data the success rate for vault suspension after hysterectomy was 100% for mild prolapse and 91.2% for advanced prolapse [1,37,48].

### 5.2. Mesh-Augmented Vaginal Repairs

Primarily, it is important to distinguish transvaginal mesh-augmented repairs from abdominal or minimally invasive sacrocolpopexy, as these procedures differ substantially in terms of indications, efficacy, and complication profiles [19].

Transvaginal mesh (TVM) was introduced more than three decades ago to reinforce weakened pelvic support structures and improve the anatomical durability of prolapse repair. Early studies reported anatomical success rates approaching 95%, particularly in women with advanced or recurrent pelvic organ prolapse in whom native tissue repair had failed [14]. An additional advantage was the possibility of addressing multiple prolapsed compartments through a single vaginal approach, often with shorter operative times and faster postoperative recovery. However, reported outcomes have varied considerably according to the mesh system used, the compartment treated, the definition of treatment success, and the duration of follow-up. Overall, the available evidence indicates that sacrocolpopexy has demonstrated more consistent long-term anatomical outcomes than transvaginal mesh repair [20,37].

Unlike native tissue reconstruction, TVM is associated with a distinct spectrum of procedure-specific complications. Reported adverse events include vaginal mesh exposure or erosion (4–40%), injury to adjacent pelvic organs, vaginal bleeding or discharge, infection, deep fascial necrosis, bladder and bowel dysfunction, fistula formation, dyspareunia, impaired sexual function, and chronic pelvic, vaginal, or lower-limb pain. In some cases, these complications require repeat surgery or mesh excision and, rarely, may be associated with severe systemic inflammatory reactions [18,49]. Although recent studies have reported satisfactory symptom improvement in certain patients, mesh-related complications continue to account for reoperation rates ranging from 5% to 15% [49,50].

These safety concerns prompted regulatory actions by several authorities, including the U.S. Food and Drug Administration (FDA), resulting in the withdrawal of transvaginal mesh kits for pelvic organ prolapse repair from the U.S. market in 2019 [18,49]. Currently, following regulatory actions of FDA, the use of transvaginal mesh has been substantially restricted or completely discontinued in many countries worldwide. Where its use remains permitted, it is generally reserved for carefully selected cases in which the benefits outweigh the risks [14,18]. Still, it should be properly used according to strict indications and performed only by trained urogynecological surgeons in specialized referral centers, in accordance with local regulatory requirements and professional guidelines. Selected older menopausal patients with recurrent or advanced multicompartment prolapse involving severe urinary incontinence, may still be considered candidates in jurisdictions where this approach remains available [20,50]. Conversely, most authors generally do not advise TVM in patients who had previous hysterectomies due to the shortened vagina, which makes application difficult and increases the complication rate [18,49].

Given the current regulatory landscape and the availability of effective mesh-free alternatives, transvaginal mesh should no longer be considered a routine reconstructive option for post-hysterectomy vaginal vault prolapse. When its use is contemplated, careful patient selection, comprehensive preoperative counseling, and treatment in experienced referral centers remain essential. For asymptomatic women with previously implanted vaginal mesh, routine clinical follow-up remains appropriate, and no additional interventions are currently recommended in the absence of mesh-related complications [18,49,50].

### 5.3. Abdominal, Laparoscopic, and Robotic Sacrocolpopexy

Sacrocolpopexy is widely considered to be the most durable surgical option for apical prolapse, with success rates of 90–95%. Using synthetic mesh implantation, the vaginal apex is attached to the anterior longitudinal ligament of the sacrum. This provides strong vertical suspension and restores anatomical alignment [1,2,51]. Systematic reviews demonstrate lower recurrence rates (often ≤10%) compared to vaginal reconstructive procedures as well as a reduced need for repeated surgery compared to native tissue techniques [25,40]. These findings explain why sacrocolpopexy is often considered to be the reference standard for long-term anatomical support. However, when recurrence is defined by symptomatic or patient-reported outcomes rather than by strict anatomical criteria, the differences between vaginal vault prolapse treatments become less pronounced [52]. Mesh can be inserted through an open or laparoscopic approach, and the route of insertion does not seem to impact its efficacy according to the literature data [53]. Laparoscopic and robotic approaches offer similar success, with shorter hospitalization and faster recovery than open surgery [54]. Their advantages include excellent long-term effects, reduced anterior compartment recurrence, and restoration of the physiological vaginal axis. Nevertheless, their disadvantages include longer operative times, higher costs, the risk of bowel or vascular injury, and mesh-related complications, such as erosion, infection, and dyspareunia. Mesh exposure rates between 3% and 10% have been reported even with abdominal placement [55,56]. Sacrocolpopexy is thus often preferred for younger sexually active women who seek maximal durability, but also for women with high recurrence risk (obesity, advanced prolapsed). Additionally, sacrocolpopexy is suggested during the course of an initial hysterectomy that is performed through a minimally invasive approach [18,55].

Abdominal uterosacral ligament suspension represents a mesh-free abdominal approach for restoration of apical support. It is usually performed at the completion of abdominal hysterectomy or during open abdominal prolapse repair by suturing the vaginal cuff to the uterosacral ligaments. This technique restores native apical support and may be preferred in patients with well-defined uterosacral ligaments, particularly when avoidance of mesh is desired. Because of the close anatomical relationship between the utero-sacral ligaments and the ureters, bilateral ureteral identification and cystoscopic confirmation of ureteral patency are important safety steps. Potential complications include ureteral kinking or injury, vaginal shortening, postoperative dyspareunia, recurrence, and sacral plexus nerve irritation [25,57].

### 5.4. Obliterative Procedures

Colpocleisis provides effective symptom relief through the obliteration of the vaginal canal, which repositions the bulging apex inside the vaginal cavity and eliminates the prolapse. This procedure is characterized by its short operative time, minimal postoperative pain, rapid recovery, low complication rates, and stable outcomes. The patients are mostly satisfied with its effectiveness as well as the improvements in their daily functioning and psychological well-being, even long-term. The success rate for further POP prevention ranges from 60% to 90%. Recurrence is rare, and operative morbidity for bladder or bowel injuries is minimal. The main complications after colpocleisis are the persistent or de novo occurrence of bladder and bowel symptoms. However, as this approach permanently eliminates vaginal intercourse, it is performed less often. It is typically applied for older or non-sexually active patients who generally face high risks for extensive surgical procedures and who give informed consent for this procedure [13,58,59].

### 5.5. Novel Techniques

Transvaginal natural orifice transluminal endoscopic surgery (vNOTES) sacrocolpopexy is an advanced scarless minimally invasive procedure that uses transvaginal access for endoscopic mesh fixation to the sacrum. By combining the advantages of vaginal surgery and laparoscopy, it offers a less invasive approach while maintaining excellent visualization of deep pelvic structures [37,60]. However, the procedure is technically demanding, requires advanced surgical expertise, and therefore remains limited to specialized centers. Still, a study showed that trained urogynecologists can learn this technique in a short period of time [60,61]. Compared with conventional laparoscopy, early studies have demonstrated reduced postoperative pain, lower analgesic requirements, shorter hospital stays, faster recovery, and excellent cosmetic outcomes. Improved visualization of critical pelvic anatomy, including the middle sacral vessels, may also contribute to a lower risk of intraoperative complications. Conversely, this technique can be challenging in patients with severe pelvic adhesions [62]. A study showed that after five years of follow-up the anatomical cure rate was 91%, while mesh exposure occurred in 3.8% of patients. Authors report recurrence rates up to 17%. Nevertheless, additional studies are needed to establish its long-term efficacy and safety [60,61,62,63]. Beyond sacrocolpopexy, the vNOTES approach has also been successfully applied to other apical prolapse procedures. Although these initial experiences are encouraging, current evidence remains limited, and larger prospective comparative studies with long-term follow-up are required before these techniques can be widely adopted [64]. At present, the available evidence is derived primarily from early clinical experience and relatively small patient series. Therefore, vNOTES should still be regarded as an emerging technique rather than an established standard for the surgical management of post-hysterectomy vaginal vault prolapse [37,61].

Pectopexy, also known as iliopectineal fixation, is a newer minimally invasive technique for apical prolapse repair in which the vaginal apex or cervix is suspended bilaterally to the iliopectineal (Cooper’s) ligaments using mesh [37,65]. A mesh-less option using monofilament non-absorbable sutures was also described [66]. Unlike sacrocolpopexy, this approach avoids dissection at the sacral promontory and may therefore reduce the risk of presacral bleeding, sacral nerve injury, and defecatory dysfunction. The mesh follows a more lateral anatomical course and does not narrow the pelvic outlet, which may be particularly advantageous in obese women or in patients with difficult access to the sacral promontory [65]. Although early evidence suggests high cure rates (over 95%) recurrence rates comparable to those of laparoscopic sacrocolpopexy (approximately 5.5%) and possibly fewer bowel-related symptoms, while chronic pain was registered in 11% of patients, long-term comparative studies are still needed before pectopexy can be widely recommended as a standard alternative [66,67]. Until more robust comparative evidence becomes available, the choice between pectopexy and established reconstructive procedures should be guided by surgeon expertise, patient anatomy, and individual clinical circumstances [37].

Robotic-assisted surgery has become increasingly relevant in urogynecology, particularly for complex pelvic reconstructive procedures requiring precise dissection, suturing, and visualization of deep pelvic structures [15,68]. The principal advantages of robotic systems include three-dimensional high-definition visualization, improved depth perception, tremor filtration, articulated instruments with an increased range of motion, enhanced ergonomics, and more precise intracorporeal suturing. In pelvic floor reconstruction, robotic assistance has been used most frequently for sacrocolpopexy, uterosacral ligament suspension, hysterectomy for benign disease, myomectomy, and selected complex fistula repairs. Robotic sacrocolpopexy may be particularly beneficial in women with severe post-hysterectomy adhesions or complex pelvic anatomy, where improved visualization facilitates meticulous dissection while reducing blood loss and postoperative pain [69]. However, its wider implementation remains limited by higher costs, longer operating room setup times, the need for specialized training, the absence of tactile feedback, and dependence on equipment availability. Therefore, robotic surgery should be considered an additional minimally invasive option for selected patients treated in experienced centers rather than a universally superior approach. Current evidence suggests that the principal advantages of robotic surgery are technical rather than clinical, as long-term anatomical and functional outcomes appear largely comparable with those achieved by conventional laparoscopic sacrocolpopexy [70,71].

### 5.6. Alternative Conservative Management

In case that surgery is contraindicated or unavailable conservative management should be considered. If adequately fitted, pessaries may offer temporary or long-term POP symptom control [72,73,74,75,76]. Pessaries are made from non-allergenic durable materials, such as silicone or poly-carbonate, and they come in different shapes and sizes, which makes them widely usable. They are usually the treatment of choice for patients who do not wish to undergo surgical procedures or who have high surgical risks as well as those with short vaginal length due to previous vaginal surgery. Pessaries are classified as support (the most common ring-shaped pessary) and space filling (suggested for advanced POP stages) [72]. Pessaries are safe and effective, exhibiting a 70–90% reduction in POP and a 50% decrease in urinary symptoms. On the other hand, they are insufficiently effective for spacious vaginas, where chance of slippage is high. Moreover, they rarely treat urinary incontinence. The literature data show that up to 50% of patients eventually undergo surgical POP corrections, mostly during the first year of use [73]. In addition hygiene of the pessaries need to be regularly checked as the most common complications are infections that can lead to vaginal ulcerations in retained pessaries and even to vaginal cancers in extreme cases [74]. Pessary treatment is dismissed mostly due to expulsion, discomfort, pain, recurrent infections, vaginal wall erosions, and continued urinary problems. Prior prolapse surgery and hysterectomy are also risk factors for ineffective pessary treatment. Advanced vault prolapse often results in poor pessary retention and limited satisfaction, with failure rates approaching 50–70%. Therefore, for women with post-hysterectomy vaginal vault prolapse, space-filling pessaries are advisable [75,76].

## 6. Functional Outcomes and Quality of Life

The decision criteria for vaginal vault prolapse surgery are traditionally based on anatomical abnormalities and/or the need for reoperation due to complication or relapse. However, recently it has been proposed that symptom relief, overall quality of life, occupational functioning, and social and sexual functioning should also be taken into consideration. Patients’ quality of life and general well-being prior to surgery can be assessed using several patient-reported outcome measures. Predictors of changes in quality of life after POP treatment could be used to identify good candidates for corrective surgery. This multidimensional approach is essential, as anatomical success does not always correlate with patient satisfaction [3,6,77,78].

While the anatomical success rates are generally comparable, perioperative morbidity differs significantly among various procedures [1,2]. Vaginal native tissue repairs typically involve shorter operative times, reduced blood loss, and faster recovery times compared to abdominal or minimally invasive sacrocolpopexy. Hospital stays are often shorter, and procedures may be performed under regional anesthesia. This makes these procedures especially suitable for elderly or medically fragile patients. Whether it is open or minimally invasive, sacrocolpopexy requires a longer operative time and carries increased risks of bowel, vascular, and sacral complications. However, in experienced hands, its overall complication rates remain low [33,34].

Improvements in prolapse-related symptoms and quality of life are consistently observed after surgery generally and across all reconstructive techniques [5,77]. Studies evaluating patient-reported outcomes show substantial reductions in bulging sensation, pelvic pressure and pain, urinary symptoms, and bowel dysfunction [3]. Currently, additional therapies are also available that can enhance the effectiveness of classic POP treatments and improve quality of life even further [79,80].

The preservation of vaginal length and sexual function represents an important consideration [81]. Vaginal native tissue repairs, including SSLF in particular, have been associated with satisfactory postoperative sexual function and minimal dyspareunia in most patients [13,16]. Compared to unilateral fixation, bilateral SSLF may further reduce postoperative discomfort and improve coital outcomes [40]. Although sacrocolpopexy is better at maintaining vaginal axis and length, sexual function outcomes appear to be broadly similar in both the abdominal and vaginal approaches according to most studies [13,81].

Reoperation represents a clinically meaningful endpoint both for patients’ quality of life and satisfaction as well as for the healthcare system. Population-based data indicate repeat surgery rates of approximately 10–20% following vaginal native tissue repairs are somewhat lower than following sacrocolpopexy [2,34]. Systemic reviews conclude that while sacrocolpopexy provides the most durable anatomical correction, native tissue vaginal repairs offer similar improvement with fewer severe complications and shorter recovery times. Differences in outcomes may thus be less clinically significant than previously assumed. Further multicenter studies on large samples are still needed to clarify this issue [1,25,33].

## 7. Clinical Decision-Making in the Surgical Management of Post-Hysterectomy Vaginal Vault Prolapse

### 7.1. Patient-Related Factors

The choice of surgical technique should begin with a comprehensive assessment of the individual patient [82]. An important factor for surgical decision-making is patient’s age. It generally reflects life expectancy, vitality and functioning, the burden of comorbidities, and overall perioperative risk. In younger women, long-term anatomical durability, preservation of vaginal anatomy, and maintenance of sexual function are frequently the primary objectives. In contrast, for older women who are no longer sexually active or who have substantial comorbidities, less invasive procedures offering shorter operative times and faster recovery may be more appropriate [83,84].

Equally important are the patient’s viewpoints and treatment goals. While some women prioritize minimizing the risk of recurrence, others choose shorter recovery period or avoiding the implantation of synthetic mesh. Consequently, individual preferences of patients must be initially discussed during the decision-making process [85].

### 7.2. Disease-Related Factors

The anatomical characteristics of the prolapse play a central role in determining the optimal surgical approach. Isolated apical prolapse differs substantially from prolapse involving the anterior and/or posterior compartments, whereas recurrent prolapse following previous reconstructive surgery often requires a different therapeutic strategy. Associated pelvic floor disorders, including stress urinary incontinence and voiding or defecatory dysfunction, should also be carefully assessed. In selected patients, simultaneous correction of these conditions may improve functional outcomes and reduce the likelihood of subsequent surgical interventions [85,86,87].

### 7.3. Procedure-Related Factors

Current evidence indicates that no single surgical procedure provides superior outcomes across all aspects of treatment. Abdominal and minimally invasive sacrocolpopexy generally achieve excellent long-term anatomical results but are associated with greater technical complexity, longer operative times, and procedure-specific complications related to the abdominal approach and mesh implantation [2,24,33]. Conversely, vaginal reconstructive procedures represent a less invasive alternative, particularly for women with increased operative risk. Although some studies have reported higher recurrence rates following certain vaginal techniques, functional outcomes, patient satisfaction, and quality of life are often comparable with those achieved after abdominal procedures [1,25,88].

Robotic sacrocolpopexy may be particularly advantageous in obese patients, in whom enhanced instrument articulation, three-dimensional visualization, and improved ergonomics may facilitate pelvic dissection and help overcome some of the technical limitations associated with conventional laparoscopy [89].

For this reason, the choice of surgical technique should not rely solely on anatomical success but should instead balance long-term efficacy, safety, potential complications, and functional outcomes [86,87].

### 7.4. Surgeon-Related Factors

The outcomes of reconstructive pelvic surgery depend not only on the selected procedure but also on surgeon experience and institutional expertise. This is particularly evident for laparoscopic and robotic sacrocolpopexy, both of which are associated with a substantial learning curve, resulting in considerable variation in outcomes across different centers. Accordingly, the choice of surgical technique cannot be separated from the expertise of the operating surgeon and the resources available within the treating institution. A procedure routinely performed with excellent results in one center may not achieve comparable outcomes in a setting with more limited experience [82,90].

### 7.5. Shared Decision-Making

Shared decision-making represents one of the fundamental principles of contemporary urogynecological surgery. Following a thorough discussion of the expected benefits, potential complications, risk of recurrence, and available treatment alternatives the final treatment decision should be guided not only by the best available evidence but also by the patient’s values, expectations, and personal priorities. Such an approach not only improves patient satisfaction but also promotes realistic expectations and a better long-term alignment between surgical outcomes and the goals that were established before treatment [82,86,91].

### 7.6. Final Considerations

Based on the currently available evidence, optimal outcomes of surgical management of post-hysterectomy vaginal vault prolapse are achieved through an individualized selection of the surgical approach based on patient characteristics, potential risks, anatomical findings, expected functional outcomes, surgeon experience, and patient preferences. This personalized treatment represents the foundation of modern urogynecological surgery and offers the best opportunity to achieve an optimal balance between anatomical correction, functional recovery, and long-term quality of life. Multicenter randomized controlled trials that would in depth compare different surgical techniques (native tissue repair, minimally invasive mesh-based procedures, emerging approaches such as vNOTES and robotic surgery) in terms of long-term durability, patient-reported outcomes, and cost-effectiveness should be undertaken in future to give more thorough information on surgical decision-making process [1,2,37,78].

**Chart 2:**
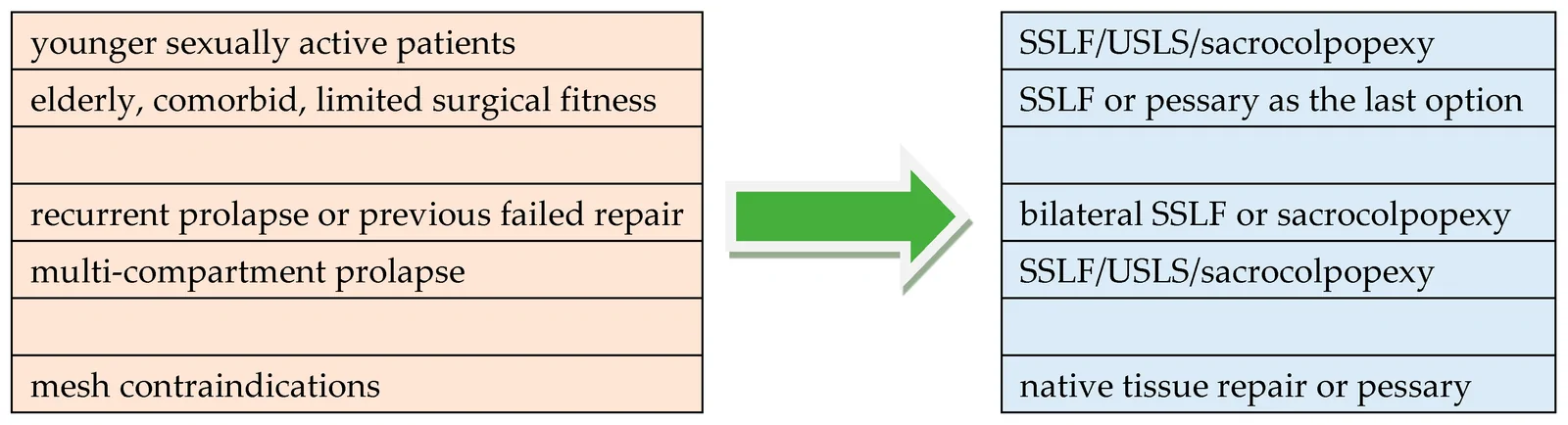
Procedure is suggested by the study authors in different clinical scenarios.

**Chart 3:**
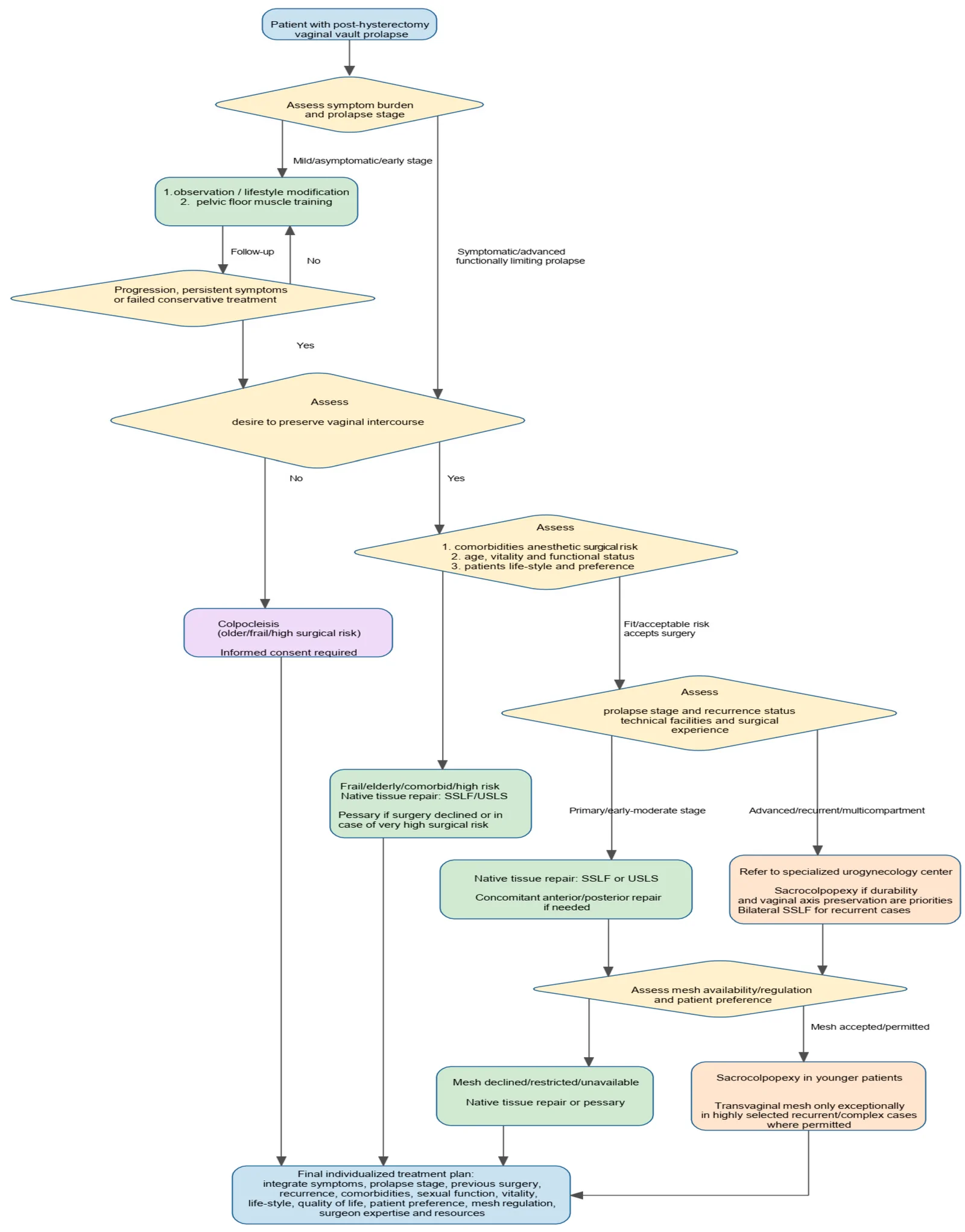
Decision-making algorithm for post-hysterectomy vaginal vault prolapse management.

The above discussed key factors influencing individualized surgical decision-making in women with post-hysterectomy vaginal vault prolapse are summarized in Table 2. Moreover, we present the practical framework showing which procedure is used by the study authors in everyday clinical work specific clinical scenarios (Chart 2). To address the complexity of treatment selection, we also propose a clinically oriented decision-making algorithm for post-hysterectomy vaginal vault prolapse management (Chart 3). Both are intended as a pragmatic clinical decision aids used by the study authors and should not be interpreted as universally applicable and validated guideline. Finally, study authors point out that native tissue repair (SSLF) had excellent outcomes for our patient so far and therefore is the treatment of choice for all of our patients.

## 8. Discussion

The present review synthesizes contemporary evidence regarding surgical management of post-hysterectomy vaginal vault prolapse. It also highlights several clinically relevant observations out of which the most important is the fact that the selection of exact surgical technique remains challenging because published data are heterogeneous, direct comparisons between procedures are limited, and decision-making often depends on recurrence status, prolapse extent, patient comorbidities, sexual activity, frailty, surgical preference and expertise. In this context, native tissue vaginal repairs, sacrocolpopexy, pessary treatment, and obliterative procedures should be regarded as complementary rather than competing options, with the optimal choice depending on the clinical scenario and patient priorities [33,88,92].

The restoration of apical support remains the cornerstone of successful prolapse re-pair. Anatomical studies consistently demonstrate that the failure of Level I support is the primary mechanism underlying vault eversion. Neglecting apical correction significantly increases recurrence rates after isolated anterior or posterior repairs. This principle explains why modern reconstructive strategies universally incorporate some form of apical suspension [24,25].

Although abdominal sacrocolpopexy is frequently the therapy of choice due to its superior anatomical durability, the clinical relevance of this advantage should be interpreted cautiously. Sacrocolpopexy demonstrates lower recurrence rates in several comparative studies [17,52]. However, patient-reported symptom improvement and quality-of-life outcomes appear to be broadly similar in both the abdominal and vaginal native tissue approaches. From a patient-centered perspective, the functional recovery and complication risks may thus be more important than purely anatomical endpoints [77,78].

Native tissue vaginal repairs, such as SSLF and USLS, seem to present an optimal balance between efficacy, safety, and recovery. These procedures are allied with shorter operative times, lower perioperative morbidity, and feasibility under regional anesthesia. These outcomes are especially advantageous for patients who are elderly or have numerous comorbidities. The long-term outcomes reported for SSLF confirm durable symptom relief with acceptable recurrence rates that are comparable to many abdominal approaches [16,36,44,45].

The decline in transvaginal mesh use has further reinforced this shift toward native tissue surgery. Transvaginal mesh application initially promised improved durability. However, subsequent reports of erosion, dyspareunia, chronic pain, and reoperation led to regulatory restrictions and substantial reconsideration of routine transvaginal mesh use. Current practices increasingly favor cautious and selective rather than routine application of synthetic materials [18,19,20].

Nevertheless, the available evidence should be interpreted with caution. Many reports are based on retrospective cohorts, small single-center series, or inadequately powered comparative studies, while selective outcome reporting and publication bias cannot be excluded. The current literature is limited by heterogeneity in study design, variable definitions of anatomical success, recurrence, and treatment failure, as well as inconsistent reporting of functional and quality-of-life outcomes. In addition, follow-up duration differs considerably across studies, and many reports are based on retrospective cohorts or single-center experiences rather than adequately powered comparative trials. As a result, direct comparison between procedures remains difficult, and apparently conflicting results across studies may partly reflect methodological differences rather than true differences in efficacy [1,2,3,88,92].

Another important limitation of the available evidence is that many comparative studies continue to prioritize anatomical correction as the primary endpoint, whereas patient-reported outcomes, functional recovery, sexual function, and long-term treatment satisfaction are reported inconsistently. As a result, superior anatomical outcomes do not necessarily translate into superior overall clinical benefit. Future comparative studies should incorporate standardized patient-reported and functional outcome measures alongside anatomical success to better inform individualized surgical decision-making [1,2,3,88,92].

Importantly, treatment selection should not be uniform. Younger sexually active women with long life expectancies may benefit from sacrocolpopexy due to its lower long-term anatomical failure rates. Conversely, older or frail patients often prioritize shorter recovery and lower surgical risk, thus making vaginal native tissue repairs preferable. For women who are not sexually active, obliterative procedures remain highly effective and minimally invasive [25,33,81].

Finally, it should be pointed out that in complex or recurrent cases, treatment planning should ideally be discussed within a multidisciplinary urogynecological and pelvic floor team. Such approach allows integration of urological, gynecological, general surgical, anesthesiology and, when needed, colorectal expertise, while also considering available facilities, mesh regulations, and the surgeon’s experience for proposed procedure. Whenever possible, management should be conducted in highly specialized tertiary centers with an established urogynecological multidisciplinary board, ensuring consensus-based decision-making and optimal selection of the safest and most effective approach for each patient [90,91].

Another challenge in interpreting the available evidence is the lack of standardized definitions of treatment success, recurrence, and failure across published studies. Differences in follow-up duration, outcome assessment, and reporting methodology further limit meaningful comparisons between surgical techniques. Future prospective multicenter studies using standardized outcome measures and reporting frameworks are needed to strengthen the evidence base and support more reliable treatment recommendations [8,9].

Taken together, these findings support an individualized approach based on a patient’s anatomy, symptoms, and occupational and social factors and preferences. This approach also accounts for general medical characteristics, such as age, body mass index, and comorbidities. Surgical success also depends on appropriate patient selection, surgeon expertise, and the chosen technique itself. These decision-making aspects are particularly relevant in recurrent and multicompartment post-hysterectomy vaginal vault prolapse, where the evidence base is even more limited and treatment decisions are frequently influenced by several factors at the same time. Therefore, although sacrocolpopexy, native tissue vaginal repairs, and obliterative procedures all have established roles, the currently available data are still insufficient to support a universally applicable treatment hierarchy for all clinical scenarios [82,86,92].

## 9. Conclusions

Post-hysterectomy vaginal vault prolapse remains one of the most challenging conditions in pelvic reconstructive surgery, not because effective surgical procedures are lacking, but because no single approach is appropriate for every patient. Current evidence supports a paradigm shift from procedure-centered to patient-centered surgical decision-making, in which anatomical findings, symptom burden, functional expectations, recurrence risk, comorbidities, patient preferences, and surgeon expertise are integrated into a single individualized treatment strategy. The next generation of clinical research should move beyond comparisons of anatomical outcomes alone and place greater emphasis on standardized functional outcomes, patient-reported outcome measures, and long-term quality of life to better define the appropriate role of individual reconstructive procedures. Ultimately, the goal is not to identify one universally superior operation but to select the most appropriate procedure for each individual patient. In the era of personalized medicine, the hallmark of successful treatment is not choosing the “best” surgical technique, but offering the right procedure to the right patient at the right time. Consequently the emphasis in surgical decision-making should be put on individualized approach chosen in collaboration between patients and surgeons.

## Figures and Tables

**Table 1:** Comparative characteristics of the principal surgical approaches for post-hysterectomy vaginal vault prolapse.

Procedure	Approximate Anatomical Success (%)	Approximate Recurrence (%)	Main Procedure-Related Complications	Recovery Profile	Typical Clinical Scenarios
Sacrospinous ligament fixation (SSLF)	80–90	10–20	Bleeding, pelvic organ injury, pudendal nerve irritation, buttock pain	Short recovery	Older women, medically comorbid patients, and those preferring native tissue reconstruction
Uterosacral ligament suspension (USLS)	75–90	10–25	Ureteral injury or kinking, bleeding	Short recovery	Women undergoing concomitant hysterectomy, those preferring native tissue reconstruction, and preservation of the physiological vaginal axis
Abdominal/laparoscopic sacrocolpopexy	90–95	<10	Mesh exposure, bowel, bladder or vascular injury, longer operative time	Longer recovery	Younger sexually active women, recurrent prolapse, and patients requiring durable long-term apical support
Transvaginal mesh-augmented repair *	75–90	5–15	Mesh exposure/erosion, chronic pelvic pain, infection, dyspareunia, pelvic organ injury	Variable recovery	Carefully selected women with recurrent or complex prolapse treated in experienced centers where the procedure is permitted

Footnote. The reported outcome ranges represent approximate estimates derived from the available literature and may vary according to study design, patient selection, duration of follow-up, surgeon experience, and definitions of treatment success. The comparison reflects the authors’ synthesis of the current evidence presented throughout this review. Accordingly, the table should be interpreted as a conceptual framework to support evidence-based, individualized surgical decision-making rather than as a prescriptive treatment algorithm. Final procedure selection should integrate patient characteristics, prolapse severity, previous surgery, expected functional outcomes, surgeon expertise, patient preferences, and local regulatory considerations regarding mesh use [1,2,25,33]. * Additional footnote. Transvaginal mesh-augmented repair has been substantially restricted or discontinued in many countries because of safety concerns. Where still permitted, its use is generally limited to carefully selected women with recurrent or complex prolapse and should be performed only by appropriately trained surgeons in experienced referral centers, in accordance with local regulatory requirements [18,19,20].

**Table 2:** Key factors influencing individualized surgical decision-making in women with post-hysterectomy vaginal vault prolapse.

Factor	Clinical Significance	Potential Impact on Surgical Decision-Making
Age and life expectancy	Reflect functional demands, perioperative risk, and the expected long-term durability of surgical repair.	Should be considered together with biological status and patient goals rather than as isolated criteria.
Sexual activity and desire to preserve vaginal function	Essential when choosing between reconstructive and obliterative procedures.	Sexually active women generally benefit from procedures preserving vaginal anatomy, whereas colpocleisis may be appropriate for carefully selected women who no longer desire vaginal intercourse.
Comorbidities and perioperative risk	Influence surgical tolerance and postoperative recovery.	Less invasive vaginal procedures may be preferable in women with substantial medical comorbidities.
Severity and compartment involvement of prolapse	Determine the anatomical complexity and the need for multicompartment reconstruction.	The surgical approach should be tailored according to the extent of apical and associated anterior and/or posterior compartment defects.
Previous pelvic floor surgery or recurrent prolapse	May increase technical complexity and the risk of recurrence.	Individualized planning is required, taking previous repairs and available reconstructive options into account.
Concomitant pelvic floor disorders	Stress urinary incontinence, voiding dysfunction, bowel dysfunction, or defecatory symptoms frequently coexist.	Simultaneous correction should be considered when clinically indicated to optimize functional outcomes.
Expected anatomical and functional outcomes	Anatomical success does not necessarily correlate with patient satisfaction.	Procedure selection should balance long-term durability, symptom relief, quality of life, and preservation of pelvic floor function.
Procedure-related risks and potential complications	Each surgical technique has distinct advantages and limitations.	Selection should reflect the balance between expected benefits and potential complications rather than anatomical success alone.
Surgeon experience and institutional expertise	Strongly influence surgical safety and treatment outcomes, particularly for minimally invasive procedures.	Procedures should preferably be performed by surgeons and centers experienced in the selected technique.
Availability of surgical resources	Access to laparoscopy, robotic platforms, specialized equipment, and multidisciplinary care varies among institutions.	The optimal procedure should also be feasible within the available healthcare setting.
Patient preferences and shared decision-making	Represent a fundamental principle of contemporary pelvic reconstructive surgery.	Final treatment selection should integrate the best available evidence with informed patient preferences, expectations, and values.

Footnote. This table summarizes the principal clinical factors that should be integrated into individualized surgical decision-making for women with post-hysterectomy vaginal vault prolapse. No single factor should independently determine procedure selection. Instead, the optimal surgical approach should result from a comprehensive assessment of patient characteristics, disease-related factors, the best available evidence, surgeon expertise, and shared decision-making.

## Data Availability

No new data were created or analyzed in this study. Data sharing is not applicable to this article.

## References

[1] Maher C., Yeung E., Haya N., Christmann-Schmid C., Mowat A., Chen Z., Baessler K. (2023). Surgery for women with apical vaginal prolapse. Cochrane Database Syst. Rev..

[2] Geoffrion R., Larouche M. (2021). Guideline No. 413: Surgical management of apical pelvic organ prolapse in women. J. Obstet. Gynaecol. Can..

[3] Segal S., Arya L.A., Smith A.L. (2012). Functional outcomes for incontinence and prolapse surgery. Curr. Bladder Dysfunct. Rep..

[4] Wu J.M., Vaughan C.P., Goode P.S., Redden D.T., Burgio K.L., Richter H.E., Markland A.D. (2014). Prevalence and trends of symptomatic pelvic floor disorders in U.S. women. Obstet. Gynecol..

[5] Rooney K., Kenton K., Mueller E.R. (2010). Distribution, risk factors, and symptom bother in women with symptomatic pelvic organ prolapse. Am. J. Obstet. Gynecol..

[6] Barber M.D., Maher C. (2013). Epidemiology and outcome assessment of pelvic organ prolapse. Int. Urogynecol. J..

[7] Persu C., Chapple C.R., Cauni V., Gutue S., Geavlete P. (2011). Pelvic Organ Prolapse Quantification System (POP-Q)—A new era in pelvic prolapse staging. J. Med. Life.

[8] Haylen B.T., de Ridder D., Freeman R.M., Swift S.E., Berghmans B., Lee J., Monga A., Petri E., Rizk D.E., Sand P.K. (2010). IUGA/ICS joint report on terminology for female pelvic floor dysfunction. Neurourol. Urodyn..

[9] Barber M.D. (2016). Pelvic organ prolapse. BMJ.

[10] Degliuomini R., Serati M., Ruffolo A.F. (2021). Evolution of untreated posterior vaginal wall prolapse. Neurourol. Urodyn..

[11] Kayondo M., Geissbuehler V., Migisha R., Kajabwangu R., Njagi J., Kato P.K. (2022). Risk factors for recurrence of pelvic organ prolapse after vaginal surgery among Ugandan women. Int. Urogynecol. J..

[12] Hammad F.T., Elbiss H.M., Osman N. (2018). The degree of bother and healthcare seeking behavior in women with symptoms of pelvic organ prolapse from a developing Gulf country. BMC Women’s Health.

[13] Fatton B., de Tayrac R., Letouzey V., Huberlant S. (2020). Pelvic organ prolapse and sexual function. Nat. Rev. Urol..

[14] Yeung E., Baessler K., Christmann-Schmid C., Haya N., Chen Z., Wallace S.A., Mowat A., Maher C. (2024). Transvaginal mesh or grafts or native tissue repair for vaginal prolapse. Cochrane Database Syst. Rev..

[15] Damiani G.R., Villa M., Falcicchio G., Bogani G., Vercellini P., Cromi A., Serati M. (2023). Robotic sacrocolpopexy with autologous fascia lata. Gynecol. Minim. Invasive Ther..

[16] Yazdany T., Wong K., Bhatia N.N. (2011). Sacrospinous ligament fixation for pelvic organ prolapse. Curr. Opin. Obstet. Gynecol..

[17] Nygaard I., Brubaker L., Zyczynski H.M., Cundiff G., Richter H., Gantz M., Fine P., Menefee S., Ridgeway B., Visco A. (2013). Long-term outcomes following abdominal sacrocolpopexy for pelvic organ prolapse. JAMA.

[18] US Food and Drug Administration (2019). Surgical Mesh for Pelvic Organ Prolapse: Safety Communication. https://www.fda.gov/medical-devices/urogynecologic-surgical-mesh-implants/surgical-mesh-pelvic-organ-prolapse.

[19] Richter L.A., Sokol A.I. (2016). Pelvic organ prolapse—Vaginal and laparoscopic mesh: The evidence. Obstet. Gynecol. Clin. N. Am..

[20] Karp D.R., Margulies R.U. (2018). Mesh in pelvic reconstructive surgery: Controversies and recommendations. Am. J. Obstet. Gynecol..

[21] Haylen B.T., Vu D. (2022). Surgical anatomy of the vaginal vault. Neurourol. Urodyn..

[22] Bonglack M., Maetzold E., Kenne K.A., Bradley C.S., Kowalski J.T. (2022). Prospective evaluation of genital hiatus. Int. Urogynecol. J..

[23] Sinex D.C.E., Bowen S.T., Kashkoush A. (2021). Establishment of a 3D anatomical coordinate system. Comput. Methods Programs Biomed..

[24] Iglesia C.B., Smithling K.R. (2017). Pelvic Organ Prolapse. Am. Fam. Physician.

[25] Maher C., Feiner B., Baessler K., Schmid C. (2016). Surgery for women with apical vaginal prolapse. Cochrane Database Syst. Rev..

[26] Ashikari A., Kadekawa K., Tokushige A. (2025). Family history and acquired risk factors for pelvic organ prolapse: A case-control study in Japan. Sci. Rep..

[27] Wu J.M., Matthews C.A., Conover M.M., Pate V., Jonsson Funk M. (2014). Lifetime risk of stress urinary incontinence or pelvic organ prolapse surgery. Obstet. Gynecol..

[28] Amin Z., El-Naggar A.K., Offiah I., Dua A., Freeman R. (2024). Systematic review and meta-analysis of the prevalence of levator ani avulsion with obstetric anal sphincter injury and its effects on pelvic floor dysfunction. Int. Urogynecol. J..

[29] Misasi G., Ozcivit Erkan I.B., Russo E., Pisacreta E., Fidecicchi T., Montt Guevara M.M. (2026). Pelvic floor dysfunction in menopause: Screening, evaluation and management. Expert Rev. Endocrinol. Metab..

[30] Chang O.H., Saldanha I.J., Encalada-Soto D., Jalloul R.J., Rozycki S., Orlando M., White A., Yang L.C., Thompson J.C., Nihira M. (2025). Associations between hysterectomy and pelvic floor disorders: A systematic review and meta-analysis. Am. J. Obstet. Gynecol..

[31] Vergeldt T.F., Weemhoff M., IntHout J., Kluivers K.B. (2015). Risk factors for pelvic organ prolapse and its recurrence: A systematic review. Int. Urogynecol. J..

[32] Schulten S.F.M., Detollenaere R.J., IntHout J., Kluivers K.B., van Eijndhoven H.W.F. (2022). Risk factors for pelvic organ prolapse recurrence after sacrospinous hysteropexy or vaginal hysterectomy with uterosacral ligament suspension. Am. J. Obstet. Gynecol..

[33] Murphy A.M., Clark C.B., Denisenko A.A., D’Amico M.J., Vasavada S.P. (2021). Surgical management of vaginal prolapse: Current surgical concepts. Can. J. Urol..

[34] Gabriel I., Kalousdian A., Brito L.G. (2021). Pelvic organ prolapse after three modes of hysterectomy. Am. J. Obstet. Gynecol..

[35] Lukanovic A., Drazic K. (2010). Risk factors for vaginal prolapse after hysterectomy. Int. J. Gynaecol. Obstet..

[36] Drain A., Escobar C., Pape D. (2020). Prolapse repair in the elderly patient: Contemporary trends and 30-day perioperative complications. Int. Urogynecol. J..

[37] Mishra V., Mishra N., Chaudhary D., Palshetkar N., Tabrez S.Z. (2020). Advances in pelvic repair and reconstructive surgeries. UroGynecology Simplified.

[38] Zhang R.R., Zhao R.H., Zhang L., Ma R.Y., Chen M.Z. (2025). Transvaginal sacrospinous ligament fixation: Efficacy and psychological outcomes. World J. Psychiatry.

[39] Shkarupa D., Kubin N., Shapovalova E., Zaytseva A. (2020). The resurrection of sacrospinous fixation: Unilateral apical sling hysteropexy. Int. Urogynecol. J..

[40] Chene G., Cerruto E., Moret S., Nohuz E. (2023). Long-term results after bilateral sacrospinous colposuspension. J. Clin. Med..

[41] Athanasiou S., Prodromidou A., Zacharakis D., Koulakmanidis A.M., Mascellino G., Douligeris A., Kathopoulis N., Grigoriadis T. (2026). Technical challenges and surgical considerations in sacrospinous ligament fixation for apical prolapse repair. J. Clin. Med..

[42] Lallemant M., Giraudet G., Delporte V., Behal H., Rubod C., Delplanque S. (2022). Long-term assessment of pelvic organ prolapse reoperation risk in obese women. J. Clin. Med..

[43] Karram M.M., Maher C. (2013). Surgery for posterior vaginal wall prolapse. Int. Urogynecol. J..

[44] Nishimura K., Yoshimura K., Hoshino K. (2019). Laparoscopic uterosacral ligament colpopexy. J. Obstet. Gynaecol. Res..

[45] Campagna G., Vacca L., Panico G., Vizzielli G., Caramazza D., Zaccoletti R., Marturano M., Granese R., Arcieri M., Cianci S. (2022). Laparoscopic high uterosacral ligament suspension vs. laparoscopic sacral colpopexy for pelvic organ prolapse: A case-control study. Front. Med..

[46] Ronsini C., Vitale C., Romeo P., Sarpietro G., Torella M., Cianci S. (2025). Laparoscopic Shull technique for uterine prolapse and risk of recurrences: A retrospective comparison with vaginal hysterectomy. Int. Urogynecol. J..

[47] Kokanalı M.K., Cavkaytar S., Aksakal O., Doganay M. (2015). McCall culdoplasty vs. sacrospinous ligament fixation after vaginal hysterectomy. Eur. J. Obstet. Gynecol. Reprod. Biol..

[48] Suh D.H., Jeon M.J. (2018). Risk factors for the failure of iliococcygeus suspension for uterine prolapse. Eur. J. Obstet. Gynecol. Reprod. Biol..

[49] Degirmenci Y., Stewen K., Dionysopoulou A., Schiestl L.J., Hofmann K., Skala C., Hasenburg A., Schwab R. (2024). Trends in urogynecology—Transvaginal mesh surgery in Germany. J. Clin. Med..

[50] Kasyan G., Abramyan K., Popov A.A., Gvozdev M., Pushkar D. (2014). Mesh-related and intraoperative complications of pelvic organ prolapse repair. Cent. Eur. J. Urol..

[51] Zhu H., Sun Y., Zheng X. (2021). Modified laparoscopic uterine suspension vs. sacrospinous fixation. Am. J. Transl. Res..

[52] Biler A., Ertas I.E., Tosun G., Hortu I., Turkay U., Gultekin O.E., Igci G. (2018). Perioperative complications and short-term outcomes of abdominal sacrocolpopexy, laparoscopic sacrocolpopexy, and laparoscopic pectopexy for apical prolapse. Int. Braz. J. Urol..

[53] Coolen A.L., van Oudheusden A.M., van Eijndhoven H.W., van der Heijden T.P., Stokmans R.A., Mol B.W., Bongers M.Y. (2013). A comparison of complications between open abdominal sacrocolpopexy and laparoscopic sacrocolpopexy for the treatment of vault prolapse. Obstet. Gynecol. Int..

[54] Pan K., Zhang Y., Wang Y., Wang Y., Xu H. (2016). A systematic review and meta-analysis of conventional laparoscopic sacrocolpopexy versus robot-assisted laparoscopic sacrocolpopexy. Int. J. Gynaecol. Obstet..

[55] Costantini E., Mearini L., Lazzeri M. (2016). Laparoscopic versus abdominal sacrocolpopexy: A randomized controlled trial. J. Urol..

[56] *PLoS ONE* Editors (2025). Editorial note: Risk factors for vaginal mesh erosion after sacrocolpopexy in Korean women. PLoS ONE.

[57] Lee W., Tam J., Kobashi K. (2019). Surgery for apical vaginal prolapse after hysterectomy: Abdominal sacrocolpopexy. Urol. Clin. N. Am..

[58] Grzybowska M.E., Futyma K., Kusiak A., Wydra D.G. (2022). Colpocleisis as an obliterative surgery for pelvic organ prolapse. Int. Urogynecol. J..

[59] Yildiz C., Ozdemir A.Z., Barutcu B., Kullac F.M., Akkafa I., Baki Erin K., Erin R. (2026). Colpocleisis as an obliterative surgery for pelvic organ prolapse: A single-center experience. Medicine.

[60] Liu J., Kohn J., Sun B., Guan Z., Liang B., Guan X. (2019). Transvaginal natural orifice transluminal endoscopic surgery sacrocolpopexy: Tips and tricks. J. Minim. Invasive Gynecol..

[61] Abiodun O., Siddiqui G., Chen H.Y., Jalloul R. (2026). vNOTES vs minimally invasive colpopexy: National study and literature review. Int. Urogynecol. J..

[62] Liu W., Sun N., Zhang Y., Li Y., Lai W., Zhou Y., Guan X., Liu J. (2025). Analyzing the learning curve of vNOTES sacrocolpopexy by the cumulative summation test: A cohort study. Int. J. Surg..

[63] Alay I., Kaya C., Cengiz H., Yildiz S., Aslan O., Yasar L., Ekin M. (2021). Apical pelvic organ prolapse repair via vaginal-assisted natural orifice transluminal endoscopic surgery: Initial experience from a tertiary care hospital. Asian J. Endosc. Surg..

[64] Issat T., Sachadel K. (2025). Vaginal natural orifice transluminal endoscopic surgery (vNOTES): A minimally invasive approach in gynecology. A comprehensive review. Ginekol. Pol..

[65] Hai P., Tian Y., Wang L., Yan S., Zhang Y., Wang Q., Zhao Q., Guo R. (2025). Clinical application of laparoscopic pectopexy in the treatment of pelvic organ prolapse: Efficacy and safety. Eur. J. Med. Res..

[66] Pirtea M., Bălulescu L., Pirtea L., Brasoveanu S., Secosan C., Balan L., Olaru F., Dabica A., Margan M.M., Navolan D. (2025). The Effectiveness of Mesh-Less Pectopexy in the Treatment of Vaginal Apical Prolapse-A Prospective Study. Diagnostics.

[67] Noe G., Farsaki E., Anapolski M., Pitsillidi A. (2026). Ten Years of Laparoscopic Pectopexy: A Case Series Analysis. Int. Urogynecol. J..

[68] Keshavamurthy M., Palshetkar N., Tabrez S.Z. (2020). Robotics in urogynecology. UroGynecology Simplified.

[69] Daykan Y., Rotem R., O’Reilly B.A. (2023). Robot-assisted laparoscopic pelvic floor surgery: Review. Best Pract. Res. Clin. Obstet. Gynaecol..

[70] Giannini A., Russo E., Malacarne E., Cecchi E., Mannella P., Simoncini T. (2019). Role of robotic surgery on pelvic floor reconstruction. Minerva Ginecol..

[71] Clifton M.M., Pizarro-Berdichevsky J., Goldman H.B. (2016). Robotic Female Pelvic Floor Reconstruction: A Review. Urology.

[72] Sansone S., Sze C., Eidelberg A. (2022). Role of pessaries in the treatment of pelvic organ prolapse. Obstet. Gynecol..

[73] Patnam R., Sripad A.A., Dengler E. (2020). How many women opt for surgery after pessary use for prolapse?. Female Pelvic Med. Reconstr. Surg..

[74] Coelho S.C.A., Giraldo P.C., Florentino J.O., Castro E.B., Brito L.G.O., Juliato C.R.T. (2017). Can the pessary use modify the vaginal microbiological flora? A cross-sectional study. Rev. Bras. Ginecol. Obstet..

[75] Van der Vaart L.R., Vollebregt A., Milani A.L. (2022). Pessary or surgery for symptomatic pelvic organ prolapse: The PEOPLE study. BJOG.

[76] Niigaki D.I., Silva R.S.P., Bortolini M.A.T., Fitz F.F., Castro R.A. (2022). Predictors for long-term adherence to vaginal pessary in pelvic organ prolapse: A prospective study. Int. Urogynecol. J..

[77] Chauvin C., Chereau E., Ballester M., Darai E. (2012). Potential relevance of pre-operative quality of life questionnaires to identify candidates for surgical treatment of genital prolapse: A pilot study. BMC Urol..

[78] Peinado Molina R.A., Hernández Martínez A., Martínez Vázquez S., Martínez Galiano J.M. (2023). Influence of pelvic floor disorders on quality of life in women. Front. Public Health.

[79] Manna P., Pecorino B., Perrone F. (2026). Topical hyaluronic acid and oxygen for urinary incontinence. Front. Med..

[80] Ruffolo A.F., Braga A., Torella M. (2022). Vaginal laser therapy for female stress urinary incontinence. Medicina.

[81] Zietarska Cisak M., Zwierzchowska A., Barcz E., Horosz E. (2023). Sexual function in women with pelvic organ prolapse and surgery influence. Ginekol. Pol..

[82] Ferrari A., Bellè N., Giannini A., Simoncini T., Vainieri M. (2024). Determinants of women’s preferences for surgical versus conservative management for pelvic organ prolapse: A survey-based study from Italy. BMJ Open.

[83] Chan S.S., Cheung R.Y., Yiu K.W., Lee L.L., Pang A.W., Chung T.K. (2012). Symptoms, quality of life, and factors affecting women’s treatment decisions regarding pelvic organ prolapse. Int. Urogynecol. J..

[84] Pecorella G., Morciano A., Sparic R., Tinelli A. (2024). Literature review, surgical decision-making algorithm, and AGREE II-S comparison of national and international recommendations and guidelines in pelvic organ prolapse surgery. Int. J. Gynaecol. Obstet..

[85] Badiu D., Onuc S., Niculescu C., Clinci D., Tica V. (2024). Treatment decision-making process among postmenopausal women with pelvic organ prolapse before gynecological surgery: A qualitative study from Romania. Medicine.

[86] Rahman S., Wallace S.L., Spivak A.R., Hortian V.A. (2025). Ways to repair multicompartment prolapse: Decision-making and surgical approach. Clin. Colon Rectal Surg..

[87] Sanses T.V., Schiltz N.K., Couri B.M., Mahajan S.T., Richter H.E., Warner D.F., Guralnik J., Koroukian S.M. (2016). Functional status in older women diagnosed with pelvic organ prolapse. Am. J. Obstet. Gynecol..

[88] Larouche M., Belzile E., Geoffrion R. (2021). Surgical management of symptomatic apical pelvic organ prolapse: A systematic review and meta-analysis. Obstet. Gynecol..

[89] Kissane L.M., Calixte R., Grigorescu B., Finamore P., Vintzileos A. (2017). Impact of obesity on robotic-assisted sacrocolpopexy. J. Minim. Invasive Gynecol..

[90] Collins S.A., Mueller M.G., Lewicky-Gaupp C., Kenton K. (2024). Surgical decision-making: Who should be offered sacrocolpopexy?. Int. Urogynecol. J..

[91] Ossin D.A., Carter E.C., Cartwright R., Violette P.D., Iyer S., Klein G.T., Senapati S., Klaassen Z., Botros S.M. (2022). Shared decision-making in urology and female pelvic floor medicine and reconstructive surgery. Nat. Rev. Urol..

[92] Padoa A., Braga A., Brecher S., Fligelman T., Mesiano G., Serati M. (2025). Pelvic organ prolapse: Current challenges and future perspectives. J. Clin. Med..

